# Risk Factors and Complication Rates of Inpatient Genioplasty Procedures in the U.S. Healthcare System: A 22-Year Retrospective Multi-center Analysis

**DOI:** 10.1007/s00266-026-06103-8

**Published:** 2026-06-19

**Authors:** Leonard Knoedler, Robert Munzinger, Benedikt Geldner, Tobias Niederegger, Emre Karakas, Simon Bigus, Alexandre G. Lellouch, Jakob Fenske, Jan O. Voss

**Affiliations:** 1 Department of Oral and Maxillofacial Surgery, Charité – Universitätsmedizin Berlin, Corporate Member of Freie Universität Berlin, Berlin Institute of Health, Humboldt-Universität zu Berlin, Berlin, Germany; 2 Department of Plastic, Reconstructive and Aesthetic Surgery, Cedars Sinai Hospital, Los Angeles, CA USA; 3 Medical Faculty, University of Heidelberg, Heidelberg, Germany; 4 Department of Oral and Maxillofacial Surgery, RWTH Aachen University, Aachen, Germany; 5 Inserm The Paris Cardiovascular Research Center, Team Endotheliopathy and Hemostasis Disorders, Université Paris Cité, Paris, France; 6 Hematology Department, AP-HP, Hôpital Européen Georges Pompidou, Paris, France

**Keywords:** Genioplasty, Facial surgery, HCUP-NIS, Aesthetic surgery, Surgical outcomes, Surgical complications

## Abstract

**Background:**

Nationwide U.S. data describing inpatient genioplasty outcomes are limited, particularly regarding complication prevalence and predictors. Leveraging HCUP-NIS, this study characterized inpatient complication patterns by procedural approach, age, and socioeconomic markers.

**Methods:**

We conducted a retrospective HCUP‑NIS analysis (2000–2022) of inpatient genioplasty admissions, evaluating demographic, socioeconomic, procedural (osseous vs. non‑osseous), hospitalization (length of stay, charges, discharge), and inpatient‑coded complication variables, with subgroup comparisons by age, income quartile, and procedure type. Stratified univariate and multivariate logistic regression identified independent correlates of inpatient-coded complications.

**Results:**

The cohort included 485 inpatient genioplasty admissions (mean age 31.8 years; 53.4% female), predominantly privately insured and residing in higher income quartiles. Complications occurred in 10.3% (n = 50), and were higher in older vs. younger patients (14.8% vs. 5.8%; *p* = 0.002). Osseous genioplasty comprised 63.5% of admissions and showed longer hospitalization, higher charges and higher complication rates versus non-osseous procedures (12.7% vs. 6.2%; *p* = 0.04). Non‑elective admission, length of stay, and primary payer were independent predictors of complications in the osseous subgroup, whereas in the non‑osseous subgroup, length of stay was the only independent predictor (*p* < 0.0001).

**Conclusion:**

In a nationwide analysis of 485 inpatient genioplasty admissions, complications occurred in ~ 10% of patients, concentrated among older, more comorbid individuals. Prolonged length of stay was the most consistent correlate of complications across techniques, while non-elective admissions increased complication risk and resource use in the osseous subgroup. Lower-income patients experienced longer hospitalizations despite similar complication rates, highlighting persistent socioeconomic disparities. Future, larger-scale prospective trials are warranted to confirm our findings.

**Level of Evidence III:**

Prognostic study. This journal requires that authors assign a level of evidence to each article. For a full description of these Evidence-Based Medicine ratings, please refer to the Table of Contents or the online Instructions to Authors www.springer.com/00266.

**Supplementary Information:**

The online version contains supplementary material available at https://doi.org/10.1007/s00266-026-06103-8.

## Introduction

Facial plastic surgery has seen a steep increase over the past 15 years, with registered procedures rising to over 6.5 million in 2023, representing more than a 100% increase compared to 2009 [1]. Within this expanding field, genioplasty procedures have gained substantial popularity, driven by increasing demand for combined aesthetics and function and catalyzed by advances in virtual surgical planning [2–6].

As the most dominant portion of the lower third of the face, the chin plays a pivotal role in perceived facial balance and aesthetic perception [6, 7]. Its projection, shape, and symmetry significantly contribute to the overall facial profile and are essential in defining attractiveness, gender identity, and even personality traits [8–11].

Genioplasty can be performed either in a standalone manner or as concurrent procedure to orthognathic surgery or gender affirming care. Surgical techniques can be broadly divided into osseous genioplasty, typically involving sliding osteotomies allowing multi-directional skeletal repositioning and non-osseous techniques relying on alloplastic materials (e. g., Medpor, silicone, Proplast, and PEEK) to enhance chin projection [12–16]. Previous studies have proven genioplasty to be a generally safe procedure with favorable safety profiles, with complication rates ranging below 5% for osseous-based techniques, while identifying higher complication rates and reduced longevity for non-osseous techniques [2, 17, 18].

Beyond costs and access to surgery, population-level data are also needed to clarify inpatient safety and to identify which patients are at increased risk for clinically relevant in-hospital complications. Most existing evidence on genioplasty outcomes comes from single-surgeon or single-center series, which limit generalizability and provide only limited insight into complication patterns across different procedural approaches and patient subgroups [2, 19].

The Healthcare Cost and Utilization Project National Inpatient Sample (HCUP-NIS) provides a U.S. nationwide dataset that can be leveraged to study utilization, resource use, and in-hospital adverse events in this setting. Accordingly, this study uses HCUP‑NIS data to examine inpatient genioplasty trends and to characterize complication profiles and correlates of inpatient-coded complications, while integrating procedural approach, age, and socioeconomic markers.

## Methods

### Data Source and Selection

The Agency for Healthcare Research and Quality (AHRQ) HCUP-NIS provides a comprehensive, stratified all-payer inpatient database comprising approximately 20% of all U.S. inpatient admissions and yielding nationally representative estimates when appropriate discharge weights are applied. The database was systematically queried to identify all inpatient genioplasty admissions from 2000 to 2022 using the following procedure codes: ICD-9-CM code 76.68 for admissions prior to the ICD-10 transition in October 2015, and ICD-10-PCS codes 0WU507Z (Supplement of Chin with Autologous Tissue Substitute, Open Approach) and 0WU50KZ (Supplement of Chin with Nonautologous Tissue Substitute, Open Approach) for subsequent admissions. A total of 485 admissions met all eligibility criteria and were included in the final analytic cohort. Given that all HCUP-NIS data are de-identified, consultation with the IRB at Cedars-Sinai Medical Center (Los Angeles, CA, USA) confirmed that formal review was not required and informed consent was therefore waived.

### Patient Selection

Procedural classification into osseous and non-osseous subtypes was based on the augmentation material specified in the ICD-10-PCS nomenclature: code 0WU507Z, denoting an autologous tissue substitute, was designated as osseous genioplasty (sliding osteotomy or autologous bone graft–based techniques), whereas 0WU50KZ, denoting a nonautologous tissue substitute, was designated as non-osseous genioplasty (alloplastic implant–based augmentation, e.g., silicone, Medpor, or PEEK). For admissions coded under the non-specific ICD-9-CM code 76.68, procedural subtype was inferred from concurrent procedure and diagnosis codes where feasible. Specifically, the presence of codes such as ICD-9-CM 76.91 or ICD-9-CM 76.63 in conjunction with ICD-9-CM 76.68 within the same admission was interpreted as indicative of osseous procedures, whereas code ICD-9-CM 76.92 in combination with ICD-9-CM 76.68 was considered indicative of non-osseous procedures. Cases in which subtype could not be reliably determined were retained in the overall cohort but excluded from the osseous versus non-osseous subgroup analysis. Concomitant procedures were identified through secondary ICD codes within the same admission record and captured in the number-of-procedures variable; differentiation between isolated and combined genioplasty admissions was not possible within the constraints of the database.

### Extracted Variables

For all patients who were deemed eligible for analysis the following variables were investigated: Procedure type (osseous vs. non-osseous), epidemiologic data (age, gender, ethnicity, income quartile, primary payer, patient location), hospitalization data (length of stay, ICD-9 and ICD-10 coded procedures and diagnoses, total costs, manner of discharge, and hospital location). Inpatient complications were defined at the patient level, with each patient classified as having experienced a complication if one or more adverse events were coded during the index admission. The total number of patients experiencing at least one complication, rather than the total count of discrete complication events, was used as the outcome variable in all analyses. Annual patient income quartile (Q1-4) ranges as defined by the HCUP-NIS for the years 2000–2022 are as follows: Q1 (<$32,000–$55,999), Q2 ($32,000–$70,999), Q3 ($42,000–$93,999), Q4 (>$52,000–>$94,000).

### Statistical Analysis

Data were compiled and descriptively summarized using Microsoft Excel (Version 16.98; Microsoft Corp., Redmond, WA, USA). All inferential analyses were performed in RStudio (R version 4.4.3; 2025.02.28). Continuous variables were summarized as mean ± standard deviation and compared between subgroups using a two-sample Welch’s t-test; all tests were two-sided. Categorical variables were compared between groups using Fisher’s exact test. Subgroup analyses were conducted by procedural approach (osseous vs. non-osseous), age (below vs. at or above the cohort median), and socioeconomic status (income quartiles 1–2 vs. 3–4). A significance level of α = 0.05 was applied for all statistical tests.

To assess factors associated with inpatient complications, univariate logistic regression was first performed to screen for potential predictors. Variables were subsequently entered into a multivariate logistic regression model including the following covariates: age, weekend admission, discharge quarter, elective status, sex, emergency department admission status, length of stay (LOS), and primary payer. Table 5 reports odds ratios and 95% confidence intervals for variables reaching statistical significance in at least one subgroup; non-significant variables are omitted from the table for clarity but were included in the full model. The multivariate model excluded covariates resulting in perfect separation, multicollinearity, or model non-convergence.

## Results

### Patient Demographics

The study cohort included 485 patients with a mean age of 31.8 ± 18.9 years and a median age of 23 years (IQR 17–43). Females constituted the majority of the cohort (n = 259; 53.4%). Most patients were White (n = 271; 55.9%) followed by Hispanic (n = 46; 9.5%), Black (n = 35; 7.2%), Asian or Pacific Islander (n = 20; 4.1%), Native American (n = 4; 0.1%), and other racial groups (n = 20; 4.1%). The largest share of the cohort resided in the highest ZIP-code–based income quartile (n = 182 (37.5%), whereas 77 patients (15.9%) were in the lowest quartile. Private insurance was the predominant primary payer (n = 312; 64.3%), followed by Medicaid (n = 62; 12.8%) and Medicare (n = 56; 11.5%). Most patients lived in metropolitan areas, particularly central (20.8%) or fringe counties (16.9%) of regions with populations ≥ 1 million (Table 1).

**Table 1:** Patient demographic, hospitalization, and outcome data for the overall genioplasty patient population

Variable	Total cohort (N=485)
Patient demographic data	
Age [mean years ± SD]	31.8 ± 18.9
Age [median + (IQR)]	23 (17–43)
Female	259 (53.4%)
Ethnicity	
White	271 (55.9%)
Black	35 (7.2%)
Hispanic	46 (9.5%)
Asian or Pacific Islander	20 (4.1%)
Native American	4 (0.1%)
Other	20 (4.1%)
Unknown	89 (18.4%)
ZIP income quartile	
Low (Q1)	77 (15.9%)
Mid-low (Q2)	89 (18.4%)
Mid-high (Q3)	120 (24.7%)
High (Q4)	182 (37.5%)
Unknown	17 (3.5 %)
Primary payer/insurance	
Medicare	56 (11.5%)
Medicaid	62 (12.8%)
Private insurance	312 (64.3%)
Self-pay	30 (6.2%)
No charge	1 (0.2%)
Other	23 (4.7%)
Unknown	1 (0.2%)
Patient location	
Central counties in metro areas of ≥ 1 million population	101 (20.8%)
Fringe counties in metro areas of ≥ 1 million population	82 (16.9%)
Counties in metro areas of 250,000–99,999 population	48 (9.9%)
Counties in metro areas of 50,000–249,999 population	14 (2.9%)
Micropolitan counties	18 (3.8 %)
Not metropolitan or micropolitan counties	13 (2.7%)
Unknown	209 (43.1%)
Admission and discharge data	
Length of stay [days ± SD]	3.8 ± 7.6
Length of stay [median + (IQR)]	2 (1–3)
Total charges [US-Dollar ± SD]	$67,127 ± $115,257
Total charges [median + (IQR)]	$30,710 ($19,878–$64,777)
Patient discharge	
Routine	434 (89.5%)
Transfer other (SNF, ICF, etc)	18 (3.7%)
Home health care	32 (6.6 %)
Other	1 (0.2%)
Complication, diagnosis, and procedural data	
Number of complications [number of patients]	57 (50)
Number of diagnoses [mean ± SD]	5.1 ± 5.2
Number of diagnoses [median + (IQR)]	3 (2–6)
Number of procedures [mean ± SD]	4.5 ± 3.5
Number of procedures [median + (IQR)]	4 (3–5)
Number of chronic conditions [mean ± SD]	1.2 ± 1.8
Number of chronic conditions [median + (IQR)]	1 (0–2)
1. Hypertension	51 (10.5%)
2. Nicotin abusus	36 (7.4%)
3. Obstructive sleep apnea	27 (5.6%)
4. Gastroesophageal reflux disease	27 (5.6%)
5. Asthma	24 (4.9%)
6. Hyperlipidemia	23 (4.7%)
7. Anxiety	22 (4.5%)
8. Diabetes mellitus	16 (3.3%)
9. History of nicotin abusus	13 (2.7%)
10. Depression	11 (2.3%)

*SD* Standard deviation, *IQR* Interquartile range, *SNF* Skilled nursing facility, *ICF* Intermediate care facility.

### Hospitalization Parameters

Patients had a mean length of stay of 3.8 ± 7.6 days and a median duration of 2 days (IQR 1–3). Total hospital charges averaged $67,127 ± $115,257, with a median cost of $30,710 (IQR: $19,878–$64,777). The vast majority of patients were discharged routinely (n = 434; 89.5%), while smaller proportions required home health care (n = 32; 6.6 %) or transfer to another facility such as a skilled nursing or intermediate care facility 18; 3.7%). Hospitalizations were distributed across all U.S. regions, with the highest proportions occurring in the South Atlantic (16.1%) and Pacific (15.9%) regions, followed by the Middle Atlantic (14.0%). Complications were documented in 50 patients (10.3%). Across the cohort, patients had a mean of 5.1 ± 5.2 diagnoses per admission (median 3, IQR 2–6) and 1.2 ± 1.8 chronic conditions (median 1, IQR 0–2). The most frequently reported comorbidities included essential hypertension (10.5%), nicotine abuse (7.4%), obstructive sleep apnea (5.6%), gastroesophageal reflux disease (5.6%), asthma (4.9%), hyperlipidemia (4.7%), anxiety (4.5%), and diabetes mellitus (3.3%) (Table 1).

### Subgroup Comparison of Younger and Older Patients

Compared to the overall cohort (mean age 31.8 years, 53.4% female, 10.3% complications), older patients (≥ median age; n = 243, 50.5%) deviated in several characteristics (Table 2). They were more often White (63.4% vs. overall 55.9%), Medicare-insured (23.0% vs. 11.5%), and had higher complication rates (14.8% vs. 5.8% in young, *p* = 0.002; and 10.3% overall), longer stays (5.1 vs. 2.5 days in young, *p* = 0.009; and 3.8 days overall), and greater clinical complexity (6.8 vs. 3.5 diagnoses in young, *p* < 0.001; 5.1 and overall) (Table 3).

**Table 2:** Patient demographic, hospitalization, and outcome data for the procedural subgroup analysis

Variable	Osseous	Non-osseous	*p*
N	308 (63.5%)	177 (36.5%)	
Patient demographic data			
Age [mean years ± SD]	33.9 ± 20.3	28.1 ± 15.5	0.022
Age [median + (IQR)]	26 (18–50)	22 (17–34)	
Female	166 (53.9%)	93 (52.5%)	0.741
Ethnicity			
White	166 (53.9%)	105 (59.3%)	0.611
Black	26 (8.4%)	9 (5.1%)	
Hispanic	30 (9.7%)	16 (9.0%)	
Asian or Pacific Islander	11 (3.6%)	9 (5.1%)	
Native American	3 (1.0%)	1 (0.6%)	
Other	11 (3.6%)	9 (5.1%)	
Unknown	61 (19.8%)	28 (15.8%)	
ZIP income quartile			
Low (Q1)	57 (18.5%)	20 (11.3%)	0.112
Mid-low (Q2)	60 (19.5%)	29 (16.4%)	
Mid-high (Q3)	71 (23.1%)	49 (27.7%)	
High (Q4)	110 (35.7%)	72 (40.7%)	
Unknown	10 (2.1%)	7 (4.0%)	
Primary payer/insurance			
Medicare	46 (14.9%)	10 (5.6%)	0.003
Medicaid	34 (11.0%)	28 (15.8%)	
Private insurance	188 (61.0%)	124 (70.1%)	
Self-pay	20 (6.5%)	10 (5.6%)	
No charge	1 (0.3%)	0 (0.0%)	
Other	19 (6.2%)	4 (2.3%)	
Unknown	0 (0.0%)	1 (0.6%)	
Patient location			
Central counties in metro areas of ≥ 1 million population	63 (20.5%)	38 (21.5%)	0.691
Fringe counties in metro areas of ≥ 1 million population	52 (16.9%)	30 (16.9%)	
Counties in metro areas of 250,000–99,999 population	34 (11.0%)	14 (7.9%)	
Counties in metro areas of 50,000–249,999 population	9 (2.9%)	5 (2.8%)	
Micropolitan counties	14 (4.5%)	4 (2.3%)	
Not metropolitan or micropolitan counties	7 (2.3%)	6 (3.4%)	
Unknown	129 (41.9%)	80 (45.2%)	
Admission and discharge data			
Length of stay [days ± SD]	4.4 ± 8.9	2.6 ± 4.6	< 0.001
Length of stay [median + (IQR)]	2 (1–4)	1 (1–2)	
Total charges [US-Dollar ± SD]	$78,013 ± $133,878	$48,250 ± $68,790	0.013
Total charges [median + (IQR)]	$32,812 ($20,471–$78,010)	$28,684 ($18,204–$44,655)	
Patient discharge			
Routine	265 (86.0%)	169 (97.7%)	0.006
Transfer other (SNF, ICF, etc)	14 (2.5%)	4 (0.5%)	
Home health care	28 (5.7%)	4 (1.3%)	
Other	1 (0.7%)	0 (0.5%)	
Complication, diagnosis, and procedural data			
Number of complications	39 (12.7%)	11 (6.2%)	0.0364
Number of diagnoses [mean ± SD]	5.6 ± 5.8	4.3 ± 4.0	0.096
Number of diagnoses [median + (IQR)]	3 (2–7)	3 (2–5)	
Number of chronic conditions [mean ± SD]	1.4 ± 1.8	1.0 ± 1.9	0.036
Number of chronic conditions [median + (IQR)]	1 (0–2)	0 (0–1)	
Number of procedures [mean ± SD]	4.7 ± 4.0	4.1 ± 2.4	0.939
Number of procedures [median + (IQR)]	4 (2–6)	3 (3–4)	

*SD* Standard deviation, *IQR* Interquartile range, *SNF* Skilled nursing facility, *ICF* Intermediate care facility.

**Table 3:** Patient demographic, hospitalization, and outcome data for the age-based subgroup analysis

Variable	Young	Old	*p*
N	238 (49.5%)	243 (50.5%)	
Patient demographic data			
Age [mean years ± SD]	17.3 ± 3.3	45.9 ± 16.9	< 0.001
Age [median + (IQR)]	17 (16–19)	43 (31–58)	
Female	137 (57.6%)	122 (50.2%)	0.112
Ethnicity			
White	117 (49.2%)	154 (63.4%)	0.004
Black	14 (5.9%)	21 (8.6%)	
Hispanic	32 (13.4%)	14 (5.8%)	
Asian or Pacific Islander	13 (5.5%)	7 (2.9%)	
Native American	3 (1.3%)	1 (0.4%)	
Other	12 (5.0%)	8 (3.3%)	
Unknown	47 (19.7%)	38 (15.6%)	
ZIP income quartile			
Low (Q1)	30 (12.6%)	47 (19.3%)	0.301
Mid-low (Q2)	46 (19.3%)	43 (17.7%)	
Mid-high (Q3)	60 (25.2%)	57 (23.5%)	
High (Q4)	91 (38.2%)	91 (37.4%)	
Unknown	11 (4.6%)	5 (2.1%)	
Primary payer/insurance			
Medicare	0 (0.0%)	56 (23.0%)	< 0.001
Medicaid	49 (20.6%)	13 (5.3%)	
Private Insurance	167 (70.2%)	142 (58.4%)	
Self-pay	6 (2.5%)	23 (9.5%)	
No charge	0 (0.0%)	1 (0.4%)	
Other	16 (6.7%)	7 (2.9%)	
Unknown	0 (0.0%)	1 (0.9%)	
Patient location			
Central counties in metro areas of ≥ 1 million population	39 (16.4%)	61 (25.1%)	0.854
Fringe counties in metro areas of ≥ 1 million population	39 (16.4%)	43 (17.7%)	
Counties in metro areas of 250,000–99,999 population	22 (9.2%)	26 (10.7%)	
Counties in metro areas of 50,000–249,999 population	5 (2.1%)	9 (3.7%)	
Micropolitan counties	7 (2.9%)	8 (3.3%)	
Not metropolitan or micropolitan counties	6 (2.5%)	7 (2.7%)	
Unknown	120 (50.4%)	86 (35.4%)	
Admission and discharge data			
Length of stay [days ± SD]	2.5 ± 3.7	5.1 ± 10.0	0.009
Length of stay [median + (IQR)]	2 (1–2)	2 (1–6)	
Total charges [US-Dollar ± SD]	$38,092 ± $38,614	$95,770 ± $152,775	< 0.001
Total charges [median + (IQR)]	$26996 ($19,550–$40,663)	$40,962 ($21,352–$102,360)	
Patient discharge			
Routine	236 (99.2%)	194 (93.9%)	< 0.001
Transfer other (SNF, ICF, etc)	0 (0.0%)	18 (1.7%)	
Home health care	1 (0.4%)	31 (4.0%)	
Other	1 (0.4%)	5 (0.5%)	
Complication, diagnosis, and procedural data			
Number of complications	14 (5.8%)	36 (14.8%)	0.002214
Number of diagnoses [mean ± SD]	3.5 ± 2.3	6.8 ± 6.7	< 0.001
Number of diagnoses [median + (IQR)]	3 (2–4)	4 (2–9)	
Number of chronic conditions [mean ± SD]	0.7 ± 1.1	1.8 ± 2.3	0.003
Number of chronic conditions [median + (IQR)]	0 (0–1)	1 (0–3)	
Total number of procedures [mean ± SD]	3.8 ± 3.1	5.2 ± 4.1	0.041
Total number of procedures [median + (IQR)]	3.5 (3–5)	4 (3–6)	

*SD* Standard deviation, *IQR* Interquartile range, *SNF* Skilled nursing facility, *ICF* Intermediate care facility.

### Subgroup Comparison by Income Level

Low-income patients (Q1–Q2; n = 166, 34.2%) differed substantially from high-income patients (Q3–Q4; n=302, 62.3%). Low-income patients (n = 166, 34.2%) had more Medicaid/Medicare coverage (35.0% vs. 24.3% overall), greater clinical complexity (6.0 vs. 4.8 diagnoses; 1.6 vs. 1.1 chronic conditions), and longer stays (4.5 vs. 3.1 days; *p* < 0.001 between groups), though complication rates were similar (11.4% vs. 9.6%) (Table 4).

**Table 4:** Patient demographic, hospitalization, and outcome data for the income-based subgroup analysis

Variable	Low-income (Q1 & Q2)	High-income (Q3 & Q4)	*p*
N	166 (34.2%)	302 (74.9%)	
Patient demographic data			
Age [mean years ± SD]	33.6± 20.7	31.1 ± 17.9	0.378
Age [median + (IQR)]	24 (18–51)	22 (17–42)	
Female	85 (51.2%)	167 (55.3%)	0.359
Ethnicity			
White	76 (45.8%)	192 (63.7%)	0.008
Black	20 (12.0%)	14 (4.6%)	
Hispanic	17 (10.2%)	23 (7.6%)	
Asian or Pacific Islander	6 (3.6%)	14 (4.6%)	
Native American	1 (0.6%)	3 (1.0%)	
Other	7 (4.2%)	9 (3.0%)	
Unknown	39 (23.5%)	47 (15.6%)	
Primary payer/insurance			
Medicare	27 (16.3%)	29 (9.6%)	< 0.001
Medicaid	31(18.7%)	23 (7.6%)	
Private insurance	90 (54.2%)	216 (71.5%)	
Self-pay	8 (4.8%)	20 (6.0%)	
No charge	0 (0.0%)	1 (0.3%)	
Other	10 (6.0%)	12 (4.0%)	
Unknown	0 (0.0%)	1 (0.3%)	
Patient location			
Central counties in metro areas of ≥ 1 million population	31 (18.7%)	61 (20.2%)	< 0.001
Fringe counties in metro areas of ≥ 1 million population	13 (7.8%)	69 (22.8%)	
Counties in metro areas of 250,000–99,999 population	25 (15.1%)	21 (7.0%)	
Counties in metro areas of 50,000–249,999 population	7 (4.2%)	7 (2.3%)	
Micropolitan counties	15 (9.0%)	3 (1.0%)	
Not metropolitan or micropolitan counties	13 (7.8%)	0 (0.0%)	
Unknown	62 (37.3%)	141 (46.7%)	
Admission and discharge data			
Length of stay [days ± SD]	4.5 ± 6.3	3.1 ± 2.3	< 0.001
Length of stay [median + (IQR)]	2 (1–5)	2 (1–3)	
Total charges [US-Dollar ± SD]	$76,593 ± $115,828	$48,999 ± $49,964	0.065
Total charges [median + (IQR)]	$33,765 ($21,267–$84,615)	$35,475 ($21,552–$60,159)	
Patient discharge			
Routine	143 (86.1%)	275 (91.1%)	0.233
Transfer other (SNF, ICF, etc)	7 (4.2%)	10 (3.3%)	
Home health care	16 (9.7%)	16 (5.3%)	
Other	0 (0.0%)	1 (0.3%)	
Complication, diagnosis, and procedural data			
Number of complications	19 (11.4%)	29 (9.6%)	0.6387
Number of diagnoses [mean ± NSD]	6.0 ± 5.9	4.8 ± 4.9	< 0.001
Number of diagnoses [median + (IQR)]	4 (2–8)	3 (2–6)	
Number of chronic conditions [mean ± SD]	1.6 ± 2.3	1.1 ± 1.5	0.097
Number of chronic conditions [median + (IQR)]	1 (0–2)	1 (0–2)	
Total number of procedures [mean ± SD]	5.0 ± 3.9	4.2 ± 3.2	0.012
Total number of procedures [median + (IQR)]	4 (3–6)	3 (3–5)	

*SD* Standard deviation, *IQR* Interquartile range, *SNF* Skilled nursing facility, *ICF* Intermediate care facility.

### Subgroup Comparison by Osseous versus Non-Osseous Genioplasty

In univariate analysis, several factors were significantly associated with complications in the osseous subgroup. Increasing age was associated with higher odds of complications (OR 1.04, *p* < 0.0001), as were later admission month (OR 1.17, *p* = 0.004) and non-routine discharge disposition (OR 1.38, *p* = 0.0005). Elective procedures were associated with lower complication risk (OR 0.34, *p* = 0.02). Longer length of stay (OR 1.29, *p* < 0.0001), higher number of chronic conditions (OR 1.45, *p* = 0.002), diagnoses (OR 1.22, *p* = 0.0003), procedures (OR 1.26, *p* < 0.0001), or external cause codes—representing injury/ trauma cases—(OR 2.19, *p* < 0.0001) were significantly associated with complications. Primary payer status was also significantly correlated with lower odds of complications observed for higher payer categories (OR 0.43, *p* < 0.0001). In the non-osseous subgroup, length of stay (OR 1.11, *p* = 0.009), number of diagnoses (OR 1.21, *p* = 0.04), number of external cause codes (OR 6.41, *p* = 0.002), and number of procedures (OR 1.47, *p* = 0.009) were associated with significantly more complications. In multivariate analysis, independent predictors of complications in the osseous subgroup included non-elective admission (OR 10.98, *p* = 0.006), longer length of stay (OR 1.29, *p* < 0.0001), and primary payer status (OR 0.51, *p* = 0.047). In contrast, in the non-osseous subgroup, only length of stay remained an independent predictor of complications (OR 1.22, *p* < 0.0001) (Table 5).

**Table 5:** Odds ratios of various risk factors in logistic regression in osseous versus non-osseous genioplasty

Risk-factor	Osseous (n = 308)			Non-osseous (n = 177)		
	Odds-ratio	95% CI	*p*-value	Odds-ratio	95% CI	*p*-value
Univariate analysis						
Age	1.04	1.02 to 1.05	< 0.0001	1.03	0.99 to 1.07	0.05
Admission month	1.17	1.05 to 1.31	0.004	1.07	0.89 to 1.30	0.43
Discharge disposition	1.38	1.15 to 1.65	0.0005	1.27	0.68 to 1.98	0.38
Elective (yes)	0.34	0.14 to 0.87	0.02	0.58	0.13 to 4.03	0.53
Length of stay	1.29	1.20 to 1.40	< 0.0001	1.11	1.03 to 1.21	0.009
Primary payer	0.43	0.30 to 0.62	< 0.0001	0.48	0.23 to 1.08	0.07
Number of chronic conditions	1.45	1.15 to 1.87	0.002	1.10	0.41 to 1.69	0.76
Number of diagnoses	1.22	1.10 to 1.37	0.0003	1.21	1.01 to 1.45	0.04
Number of external cause codes	2.19	1.70 to 2.85	< 0.0001	6.41	2.11 to 21.00	0.002
Number of procedures	1.26	1.13 to 1.42	< 0.0001	1.47	1.11 to 1.96	0.009
Multivariate analysis						
Elective (no)	10.98	1.89 to 62.31	0.006	3.40	0.2400 to 220.1	0.46
Length of stay	1.29	1.16 to 1.47	< 0.0001	1.22	1.120 to 1.361	< 0.0001
Primary payer	0.51	0.25 to 0.94	0.047	0.59	0.2757 to 1.114	0.13

## Conclusion

In this nationwide analysis of 485 inpatient genioplasty admissions, complications affected approximately one in ten patients, concentrating in older individuals and those with greater comorbidity burden. Age and non-elective admissions strongly predicted adverse outcomes in osseous procedures, while prolonged length of stay emerged as the most consistent predictor of complications across osseous and non-osseous techniques—representing an actionable, dynamic parameter for inpatient surveillance. Osseous cases showed higher inpatient complication rates and resource utilization, Medicare predominance, and more complex orthognathic records. Lower-income patients exhibited higher clinical complexity and extended hospitalizations despite comparable complication incidence, highlighting socioeconomic access gradients. Prospective trials targeting modifiable risk factors—age-adjusted strategies, preoperative optimization for non-elective cases, and socioeconomic barriers—are needed to guide improvements in genioplasty safety and access.

## Discussion

Using a nationwide cohort of 485 inpatient genioplasty cases, this study investigates demographic, procedural, and socioeconomic patterns and characterizes predictors of surgical, non‑surgical, and aggregate complication risk relevant to clinical decision‑making. The average patient in our study was young, female, and privately insured. Most patients lived in large metropolitan areas and were members of the highest income quartile. Genioplasty was generally associated with few complications, short hospital stays, and routine discharge, supporting the procedure’s safety when performed in appropriately selected patients [20].

### Complication Profile

Inpatient-coded complications occurred in 10.3% of genioplasty admissions, were higher in older vs. younger patients and in osseous vs. non-osseous cases, and were similar across lower- vs. higher-income quartiles despite greater clinical complexity in the lower-income subgroup. These findings support a generally favorable inpatient safety profile, comparable to the broader complications ranges of up to 18.6% across different genioplasty techniques, but exceeding the prevalence of adverse events in small-sample single-center osseous genioplasty reports (0.55–1.77%) [2, 20]. The age-associated increase in complications aligns with broader orthognathic surgery literature, where age > 40 doubled risks of postoperative complications [21]. However, because HCUP‑NIS captures only coded inpatient events, these estimates are likely weighted toward more severe in-hospital complications and may underreport minor or outpatient sequelae such as transient neurosensory deficits [14]. Clinically, these data suggest a generally favorable inpatient safety profile in appropriately selected patients, while highlighting older patients as a higher-risk subgroup that may benefit from stricter perioperative surveillance and counseling. Higher complication rates observed in osseous cases should be interpreted cautiously, as it may reflect case-mix differences in age rather than intrinsic technique-related risk. Finally, the absence of income-stratified differences in complication rates—despite higher complexity among lower-income patients—suggests that inpatient care can effectively mitigate SES-related complication risk [22].

### Predictors and Risk Factors of Postoperative Complications

 Stratified multivariate models showed that in osseous cases, non-elective admission, LOS, and primary payer status were independent predictors of postoperative complications, while in non-osseous cases, LOS was the only independent predictor of adverse events. These associations align with broader literature in which non-elective admission typically reflects higher-acuity presentations with shorter/ no windows of opportunity for preoperative health optimization, while payer status, in the context of administrative datasets, should be understood as a surrogate for broader socioeconomic and access-related factors—such as continuity of care, preoperative optimization, and health literacy—rather than as a direct biological or procedural risk factor [23]. Accordingly, the observed association between payer status and complications rather reflects systemic disparities than a discrete causal pathway. Regarding LOS, it is important to acknowledge that its relationship with complications is inherently bidirectional: while prolonged hospitalization may expose patients to additional in-hospital risks, complications themselves can equally extend LOS [21]. This duality precludes interpretation of LOS as a purely causal preoperative risk factor and is an inherent constraint of cross-sectional administrative data, where the precise temporal sequencing of events within a hospitalization cannot be established. Nevertheless, this bidirectional relationship does not diminish its clinical utility—rather, it supports its use as a dynamic marker of in-hospital trajectory for continuous surveillance throughout the treatment course, paralleling orthognathic literature linking extended LOS with higher complication burden and care intensity [24, 25].

### Procedural Differences

Osseous procedures comprised 63.5% of admissions and were associated with longer LOS, higher charges, and higher complication rates, with patients being older and more often Medicare-insured. These differences likely reflect case-mix and selection effects, as older age and Medicare insurance are consistent with previously identified risk factors for postoperative complications, compounded by the HCUP-NIS focus on inpatient cases which inherently captures a higher-acuity patient population than outpatient case-mixes [26]. External literature reporting superior safety profiles for osseous techniques derives from fundamentally different study designs and patient populations and should therefore not be directly contrasted with our findings [14]. Rather, our data characterize a distinct higher-risk inpatient demographic profile for osseous genioplasty, suggesting that this subgroup may benefit from targeted preoperative counseling and enhanced perioperative surveillance.

### Further Demographic Disparities

Stratification by income similarly indicated that lower-income patients had higher public insurance coverage, relative overrepresentation of Black and Hispanic individuals, greater clinical complexity, and prolonged LOS, despite comparable prevalence of complications. This predominance of higher-income patients in the inpatient genioplasty cohort mirrors well-described barriers to elective surgical care, which are amplified by the metropolitan concentration of specialized services and reduced access for rural populations [27, 28]. Prior work further suggests that insurer exclusions for isolated mentoplasty, authorization requirements for functional osseous procedures, and limited ambulatory access for publicly insured patients can shape who ultimately receives surgery and where it is performed [29–32]. Addressing these disparities through targeted discharge planning and resource allocation could improve care in patient groups with higher baseline complexity. Transparent coverage criteria, streamlined and cross-disciplinary orthognathic consultation processes, and expanded outpatient networks could further enhance access to genioplasty for a broader range of patients.

## Limitations

Despite its robust size, this study has several limitations. First, the HCUP‑NIS database, despite being nationally representative, only captures information recorded during inpatient admissions. Because isolated genioplasty is typically performed in an outpatient setting, the inpatient dataset is inherently biased toward cases in which genioplasty was performed alongside additional procedures or in patients with higher medical complexity requiring inpatient care. As a result, genioplasty procedures performed exclusively in outpatient settings, as well as complications managed outside the index hospitalization, are not represented. This likely biases the sample toward more complex or higher-acuity cases and may underestimate the overall complication burden, particularly for minor or delayed events. Consequently, findings should not be generalized to standard elective cosmetic genioplasty performed in outpatient settings, which represents the predominant practice context for this procedure. Additionally, the HCUP-NIS does not provide a dedicated code to differentiate isolated genioplasty from procedures performed concurrently with orthognathic or other surgery, precluding formal stratification by procedural combination.

Second, length of stay is measured at discharge and reflects the cumulative inpatient course rather than a fixed preoperative characteristic. Its relationship with complications is inherently bidirectional: prolonged hospitalization may independently contribute to adverse events, while complications themselves frequently extend LOS. This mutual dependency introduces the possibility of reverse causation and means that LOS should be interpreted primarily as a dynamic course-of-stay correlate of complications rather than a unidirectional causal risk factor.

Third, the use of ICD‑9 and ICD‑10 coding introduces the possibility of misclassification or under‑reporting of both procedures and diagnoses. Fourth, ZIP code‑level income quartiles were used to infer patient socioeconomic status and may not accurately reflect individual‑level economic conditions. Fifth, because the data are deidentified and cross‑sectional, longitudinal tracking of patients and assessment of long‑term or repeat outcomes were not possible. Sixth, detailed clinical information such as surgical indication, operative technique, surgeon’s experience and background, use of virtual planning, and implant material is not captured in HCUP‑NIS, limiting the ability to examine how these factors influence complications and resource use. Seventh, although this study represents one of the larger inpatient analyses of genioplasty to date, the absolute number of complications remains modest, which limits the statistical power and reliability of the multivariable models and subgroup comparisons. Certain associations, particularly those with wide confidence intervals, should be considered potentially unstable, and findings should therefore be interpreted as exploratory and hypothesis-generating rather than definitive. Eighth, by being a nationwide database, the data is restricted to procedures performed in the U.S. and might therefore not reflect practice in other countries with different standards. Especially for trauma/acute cases, complications might have been due to the patient’s additional diagnoses but not caused by the genioplasty *per se*. Furthermore, ethnicity data were unknown in a substantial proportion of cases across the overall cohort and subgroups, limiting the interpretability of ethnic distribution findings and precluding robust conclusions regarding racial or ethnic disparities in genioplasty outcomes.

## Supplementary Information

Below is the link to the electronic supplementary material.Supplementary file1 (DOCX 15 KB)
