# Correction: Risk Factors and Complication Rates of Inpatient Genioplasty Procedures in the US Healthcare System: A 22-Year Retrospective Multi-center Analysis

**DOI:** 10.1007/s00266-026-06255-7

**Published:** 2026-08-26

**Authors:** Leonard Knoedler, Robert Munzinger, Benedikt Geldner, Tobias Niederegger, Emre Karakas, Simon Bigus, Alexandre G. Lellouch, Jakob Fenske, Jan O. Voss

**Affiliations:** 1 Department of Oral and Maxillofacial Surgery, Charité – Universitätsmedizin Berlin, Corporate Member of Freie Universität Berlin, Berlin Institute of Health, Humboldt-Universität zu Berlin, Berlin, Germany; 2 Department of Plastic, Reconstructive and Aesthetic Surgery, Cedars Sinai Hospital, Los Angeles, CA USA; 3 Medical Faculty, University of Heidelberg, Heidelberg, Germany; 4 Department of Oral and Maxillofacial Surgery, RWTH Aachen University, Aachen, Germany; 5 Inserm The Paris Cardiovascular Research Center, Team Endotheliopathy and Hemostasis Disorders, Université Paris Cité, Paris, France; 6 Hematology Department, AP-HP, Hôpital Européen Georges Pompidou, Paris, France

**Correction to: Aesthetic Plastic Surgery** **https://doi.org/10.1007/s00266-026-06103-8**

The original online version of this article was revised to correct the presentation of the name of coauthor Alexandre G. Lellouch.

The original article has been corrected.

