# Supplementary material for: Risk Factors and Complication Rates of Inpatient Genioplasty Procedures in the U.S. Healthcare System: A 22-Year Retrospective Multi-center Analysis
Source: Aesthetic Plast Surg. 2026 Jun 19;50(16):6655–66. doi: 10.1007/s00266-026-06103-8 (PMC13529613; doi:10.1007/s00266-026-06103-8)
Supplement: Supplementary file 1 [file 266_2026_6103_MOESM1_ESM.docx]

**Supplementary Content**

**Supplementary Table 1 - Hospital division distribution**: **Overall Cohort**

| **Hospital Division** | **Total Cohort (N=485)** |
| --- | --- |
| New England | 26 (5.4%) |
| Middle Atlantic | 68 (14.0%) |
| East North Central | 31 (6.4%) |
| West North Central | 13 (2.7%) |
| South Atlantic | 78 (16.1%) |
| East South Central | 9 (1.9%) |
| West South Central | 28 (5.8%) |
| Mountain | 7 (1.4%) |
| Pacific | 77 (15.9%) |
| Unknown | 148 (30.5%) |

**Supplementary Table 2 - Hospital division distribution: Osseous vs. Non-Osseous**

| **Hospital Division** | **Osseous (n=308)** | **Non-Osseous (n=177)** | **p** |
| --- | --- | --- | --- |
|  |  |  | 0.099 |
| New England | 14 (4.5%) | 12 (6.8%) |  |
| Middle Atlantic | 42 (13.6%) | 26 (14.7%) |  |
| East North Central | 16 (5.2%) | 15 (8.5%) |  |
| West North Central | 5 (1.6%) | 8 (4.5%) |  |
| South Atlantic | 56 (18.2%) | 22 (12.4%) |  |
| East South Central | 7 (2.3%) | 2 (1.1%) |  |
| West South Central | 13 (4.2%) | 15 (8.5%) |  |
| Mountain | 3 (1.0%) | 4 (2.3%) |  |
| Pacific | 50 (16.2%) | 27 (15.3%) |  |
| Unknown | 102 (33.1%) | 46 (26.0%) |  |

**Supplementary Table 3 - Hospital division distribution: Young vs. Old**

| **Hospital Division** | **Young (n=238)** | **Old (n=243)** | **p** |
| --- | --- | --- | --- |
|  |  |  | 0.032 |
| New England | 14 (5.9%) | 12 (4.9%) |  |
| Middle Atlantic | 41 (17.2%) | 27 (11.1%) |  |
| East North Central | 12 (5.0%) | 19 (7.8%) |  |
| West North Central | 12 (5.0%) | 1 (0.4%) |  |
| South Atlantic | 52 (21.8%) | 26 (10.7%) |  |
| East South Central | 4 (1.7%) | 4 (1.6%) |  |
| West South Central | 13 (5.5%) | 15 (6.2%) |  |
| Mountain | 3 (1.3%) | 4 (1.6%) |  |
| Pacific | 40 (16.8%) | 33 (13.6%) |  |
| Unknown | 47 (19.7%) | 101 (41.6%) |  |

**Supplementary Table 4 - Hospital division distribution: Low-Income vs. High-Income**

| **Hospital Division** | **Low-Income (n=166)** | **High-Income (n=302)** | **p** |
| --- | --- | --- | --- |
|  |  |  | <0.001 |
| New England | 7 (4.2%) | 17 (5.6%) |  |
| Middle Atlantic | 14 (8.4%) | 50 (16.6%) |  |
| East North Central | 14 (8.4%) | 17 (5.6%) |  |
| West North Central | 5 (3.0%) | 8 (2.6%) |  |
| South Atlantic | 30 (18.1%) | 46 (15.2%) |  |
| East South Central | 8 (4.8%) | 1 (0.3%) |  |
| West South Central | 12 (7.3%) | 16 (5.3%) |  |
| Mountain | 0 (0.0%) | 7 (2.3%) |  |
| Pacific | 13 (7.8%) | 60 (19.9%) |  |
| Unknown | 63 (37.9%) | 80 (26.5%) |  |

*Abbreviations: Low-income = income quartiles 1–2; High-income = income quartiles 3–4; Young = below cohort median age; Old = at or above cohort median age. p-values refer to between-group comparisons within each subgroup pair.*
